# Genome analysis of quorum sensing *Cedecea neteri* SSMD04 leads to identification of its novel signaling synthase (*cneI*), cognate receptor (*cneR*) and an orphan receptor

**DOI:** 10.7717/peerj.1216

**Published:** 2015-09-01

**Authors:** Kian-Hin Tan, Jia-Yi Tan, Wai-Fong Yin, Kok-Gan Chan

**Affiliations:** Division of Genetics and Molecular Biology, Institute of Biological Sciences, Faculty of Science, University of Malaya, Kuala Lumpur, Malaysia

**Keywords:** *N*-acyl-homoserine lactone, Quorum sensing, Food microbiology, Mass spectrometry, N-butyryl-homoserine lactone, Autoinducer

## Abstract

*Cedecea neteri* is a very rare human pathogen. We have isolated a strain of *C. neteri* SSMD04 from pickled mackerel sashimi identified using molecular and phenotypics approaches. Using the biosensor *Chromobacterium violaceum* CV026, we have demonstrated the presence of short chain *N*-acyl-homoserine lactone (AHL) type quorum sensing (QS) activity in *C. neteri* SSMD04. Triple quadrupole LC/MS analysis revealed that *C. neteri* SSMD04 produced short chain *N*-butyryl-homoserine lactone (C4-HSL). With the available genome information of *C. neteri* SSMD04, we went on to analyse and identified a pair of *luxI/R* homologues in this genome that share the highest similarity with *croI/R* homologues from *Citrobacter rodentium*. The AHL synthase, which we named *cneI*(636 bp), was found in the genome sequences of *C. neteri* SSMD04. At a distance of 8bp from *cneI* is a sequence encoding a hypothetical protein, potentially the cognate receptor, a *luxR* homologue which we named it as *cneR*. Analysis of this protein amino acid sequence reveals two signature domains, the autoinducer-binding domain and the C-terminal effector which is typical characteristic of *luxR*. In addition, we found that this genome harboured an orphan *luxR* that is most closely related to *easR* in *Enterobacter asburiae*. To our knowledge, this is the first report on the AHL production activity in *C. neteri*, and the discovery of its *luxI*/*R* homologues, the orphan receptor and its whole genome sequence.

## Introduction

*Cedecea* spp. are extremely rare Gram-negative bacteria that belong to the *Enterobacteriaceae* family (Berman, 2012). The representative of the genus is lipase-positive and resistant to colistin and cephalothin. The name *Cedecea* was coined by Grimont and Grimont, from the abbreviation of the Centers for Disease Control (CDC) (Grimont et al., 1981). Originally recognized as Enteric group 15, this genus is comprised of five species, out of which only three are valid, *C. neteri*, *C. lapagei*, *C. davisae*, while the other two were not validly published and are known as *Cedecea* species 3 and *Cedecea* species 5 (Brenner et al., 2005).

*Cedecea* species 4 was named as *C. neteri* in 1982 when its clinical significance was reported (Farmer 3rd et al., 1982). *C. neteri* was also found in a patient with systemic lupus erythematosus where it led to the patient’s death (Aguilera et al., 1995). Even though it was evident that *C. neteri* can act as human pathogen, its etiology is unknown and limited studies have been conducted on *Cedecea* spp. There were cases of isolation of *Cedecea* spp. from other sources except human (Jang & Nishijima, 1990; Osterblad et al., 1999).

Bacteria demonstrate a concerted gene regulation mechanism termed ‘Quorum Sensing’ (QS) that relies on the population density of the bacteria (Fuqua, Winans & Greenberg, 1996; Miller & Bassler, 2001; Schauder & Bassler, 2001). The mechanism of QS involves the production, release, detection, and response to small diffusible molecules known as autoinducers, such as *N*-acyl homoserine lactones (AHLs) commonly employed by Gram negative bacteria (Chhabra et al., 2005; Williams et al., 2007). AHL molecules are generally characterized by the length and saturation of the acyl side chains, which can vary from 4 to 18 carbons (Pearson, Van Delden & Iglewski, 1999), as well as the R-group substitution at the third carbon (Pearson, Van Delden & Iglewski, 1999; Waters & Bassler, 2005). QS has been shown to play a role in the regulation of a wide range of phenotypes, such as antibiotic biosynthesis, biofilm formation, pathogenesis, and bioluminescence (Fuqua, Winans & Greenberg, 1996; Salmond et al., 1995; De Kievit & Iglewski, 2000; Hastings & Nealson, 1977; Bainton et al., 1992; Eberl et al., 1996).

We have recently reported the isolation of *C. neteri* SSMD04 from *Shime saba*, a Japanese cuisine that involves marinating with salt and rice vinegar, enabling the usually perishable *saba* (mackerel) to be consumed in the form of sashimi (raw fish). The complete genome of *C. neteri* SSMD04 has been sequenced and published (Chan et al., 2014). This strain was isolated in a study to investigate the role of AHL-based QS in food spoilage and food safety (JY Tan, 2014, unpublished data). As a known human pathogen, *C. neteri* has never been reported to be isolated from food source. The adaptability of the bacterium to survive and colonize the two environments is an interesting aspect to be studied. It has been known that QS regulates virulence as well as food spoilage traits in some bacteria (Passador et al., 1993; Brint & Ohman, 1995; Skandamis & Nychas, 2012; Bruhn et al., 2004). This prompted us to test *C. neteri* SSMD04 for its QS activity. The genome sequence enabled investigation of QS related genes. Meanwhile, the presence of this bacterium in oily fish suggests possible lipolytic activity, which is present in representative of the genus *Cedecea*. Its lipase activity was also tested for this reason.

In this work, we show for the first time that *C. neteri* possesses an AHL QS system and identified a novel signaling synthase gene (*cneI*), its cognate receptor (*cneR*), and an orphan LuxR-type receptor gene.

## Materials and Methods

### Sample collection and processing

*Shime saba* sashimi sample was collected from a local supermarket in Malaysia and processed within half an hour following collection. Five grams of sample was stomached (mixing of sample in a sterile plastic bag by applying forces to the outside of the bag) using Stomacher^®^ 400 circulator (Seward, West Sussex, UK) and homogenized in 50 ml of peptone water and then spread on MacConkey (MAC) agar. The culture plates were incubated overnight at 28 °C.

### Bacterial strains, media and culture conditions

*C. neteri* SSMD04, *Chromobacterium violaceum* CV026, *Erwinia carotovora* GS101 and *E. carotovora* PNP22 were maintained in Luria Bertani (LB) medium at 28 °C. *lux*-based biosensor *Escherichia coli* (pSB401) was grown in LB supplemented with tetracycline (20 µg/mL) at 37 °C. All broth cultures were incubated with shaking (220 rpm).

### Species identification of isolate SSMD04

#### 16S rDNA phylogenetic analysis

Whole genome sequencing, assembly, annotation were performed as described previously (Chan et al., 2014). 16S rRNA gene sequence of *C. neteri* SSMD04 was searched using “Genome Browser” function in RAST (Aziz et al., 2012) after automated annotation. Other 16S rRNA gene sequences of *Cedecea*. spp. were retrieved from GenBank through text search. MEGA 6.0 (Tamura et al., 2013) was used to align the sequences with ClustalW and construct a Maximum likelihood tree using 1,000 bootstrap replications.

#### Biolog GEN III microbial identification system

Microbial identification using Biolog GEN III MicroPlate (Biolog, Hayward, California, USA) was carried out according to manufacturer’s protocol. In brief, overnight culture of *C. neteri* SSMD04 grown on Tryptic Soy Agar (TSA) was used to inoculate inoculating fluid (IF) A to a cell density of 90–98% transmittance. The inoculum was then pipetted into each well of the MicroPlate (100 µL per well) and incubated at 28 °C for 24 h. The MicroPlate was then read using the machine reader and software where the wells will be scored as ‘negative’ or ‘positive’ based on the colour change due to the reduction of tetrazolium redox dyes. This ‘Phenotypic Fingerprint’ was then used to identify the bacteria by matching it against the database in the system.

### Detection of AHL production in *C. neteri* SSMD04

AHL-type QS activity of *C. neteri* SSMD04 was screened using biosensor *C. violaceum* CV026. This is performed by cross streaking *C. neteri* SSMD04 against *C. violaceum* CV026 (McClean et al., 1997). *E. carotovora* GS101 and *E. carotovora* PNP22 were used as positive and negative controls, respestively (Jones et al., 1993).

### AHL extraction

*C. neteri* SSMD04 was cultured overnight at 28 °C in LB broth (100 mL) supplemented with 50 mM of 3-(N-morpholino)propanesulfonic acid (MOPS) (pH5.5). Culture supernatant was collected by centrifugation and organic compounds were subsequently extracted twice with equal volume of acidified ethyl acetate (AEA) (0.1% v/v glacial acetic acid). The extracts were air dried and reconstituted in 1 mL of AEA, transferred into sterile microcentrifuge tubes and air dried again. The extracts were later used for detection of AHL by *lux*-based biosensor *E. coli* (pSB401) as well as triple quadrupole LC/MS.

### AHL identification by triple quadrupole LC/MS

AHL extracts were reconstituted in acetonitrile (ACN) prior to LC/MS analysis as described before (Lau et al., 2013), with slight modifications. In brief, mobile phase A was water with 0.1% v/v formic acid and mobile phase B used was ACN with 0.1% formic acid. The flow rate used was 0.5 mL/min. The gradient profile was set to: A:B 80:20 at 0 min, 50:50 at 7 min, 50:50 at 7.10 min, 80:20 at 12 min, 80:20 at 12.10 min, 20:80 at 14 min, 20:80 at 14.10 min. Precursor ion scan mode was carried out in positive ion mode with Q1 set to monitor *m*/*z* 90 to *m*/*z* 400 and Q3 set to monitor for *m*/*z* 102 which is characteristics of lactone ring moiety. ACN was also used as a blank.

### Measurement of bioluminescence

*E. coli* (pSB401) (Winson et al., 1998) was used as biosensor for the detection of exogenous short chain AHLs present in the extracts. The biosensor strain was cultured in LB broth supplemented with tetracycline (20 µg/mL). The overnight culture was then diluted to an OD_600_ of 0.1 with fresh LB broth with tetracycline. The diluted *E. coli* culture was used to resuspend the extracts from Section ‘AHL extraction,’ prior to being dispensed into a 96-well optical bottom microtitre plate. Cell density bioluminescence measurements were carried out by Infinite M200 luminometer-spectrophotometer (Tecan, Männedorf, Switzerland) over a period of 24 h. Diluted *E. coli* culture without extracts was read for normalization and sterile broth was used as negative control. The results were displayed as relative light units (RLU)/OD_495_ nm against incubation time.

### Lipase activity

The lipase activity of *C. neteri* SSMD04 was tested in a medium consisted of (in w/v) 0.05% yeast extract, 0.1% tryptone, 1% NaCl, and 1.5% agar supplemented with 0.5% (v/v) corn oil. The oil forms an opaque suspension in the agar. Bacteria culture was streaked on the agar and incubated at 28 °C for 48 h. The enzymatic activity is visualized by a halo zone formed around the colonies caused by the breakdown of lipids.

### *luxI/R* homologues search and analysis

Whole genome of *C. neteri* SSMD04 was sequenced and annotated (Chan et al., 2014). The *luxI/R* homologues were searched on RAST using the “Genome Browser” function. The “Annotation Overview” function on RAST was used to make locus comparison of *cneI/R* pair and the orphan *luxR* with other genomes. Multiple sequence alignment of LuxR-type proteins was done with ClustalW. ESPript (Robert & Gouet, 2014) was used for the presentation of the alignment.

## Results

As was mentioned in the introduction, *C. neteri* SSMD04 was isolated from *shime saba* in an attempt to recover AHL-producing bacteria from food. It was identified through 16S rRNA gene sequence and Biology Gen III microbial identification system. The novelty of this bacterium, coupled with the possible role it plays in food spoilage and known clinical aspects of other members of the same species, led to further analysis.

### Species identification of *C. neteri* SSMD04

**Figure 1:**
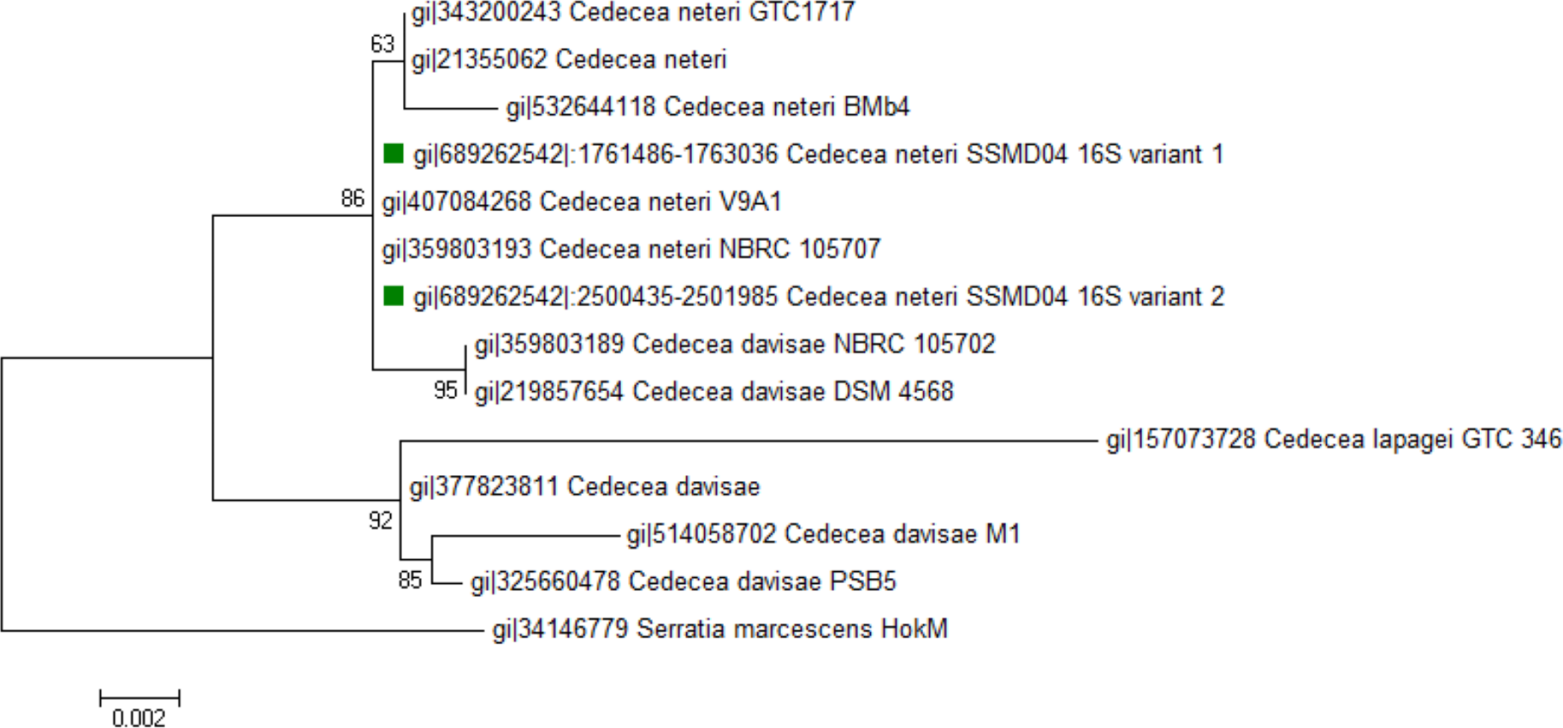
Phylogenetic tree showing the position of *C. neteri* SSMD04 (green squares) relative to other *Cedecea* spp. The maximum likelihood tree was inferred from 1,297 aligned positions of the 16S rDNA sequences using Hasegawa-Kishino-Yano substitution model. Boostrap values are represented at the nodes. The scale denotes the number of substitutions per nucleotide position. *Serratia marcescens* strain HokM was used as outgroup.

Seven 16S rRNA gene sequences were found in *C. neteri* SSMD04 genome, of which 6 were identical and the other has 2 SNPs. Both variants were included in phylogenetic analysis with other sequences of *Cedecea* spp. available in GenBank. As can be seen, 16S rRNA gene sequences of *C. neteri* SSMD04 and other *C. neteri* strains appeared in a monophyletic clade (Fig. 1).

The Biology Gen III microbial identification system was also used to assess the identity of *C. neteri* SSMD04 biochemically. The system identified this strain to be *C. neteri* with a probability and similarity of 0.697. The positive reaction in sucrose well and D-sorbitol well agrees with the report of Farmer 3rd et al. (1982).

### Detection of AHL-type QS activity in *C. neteri* SSMD04 using AHL biosensor

**Figure 2:**
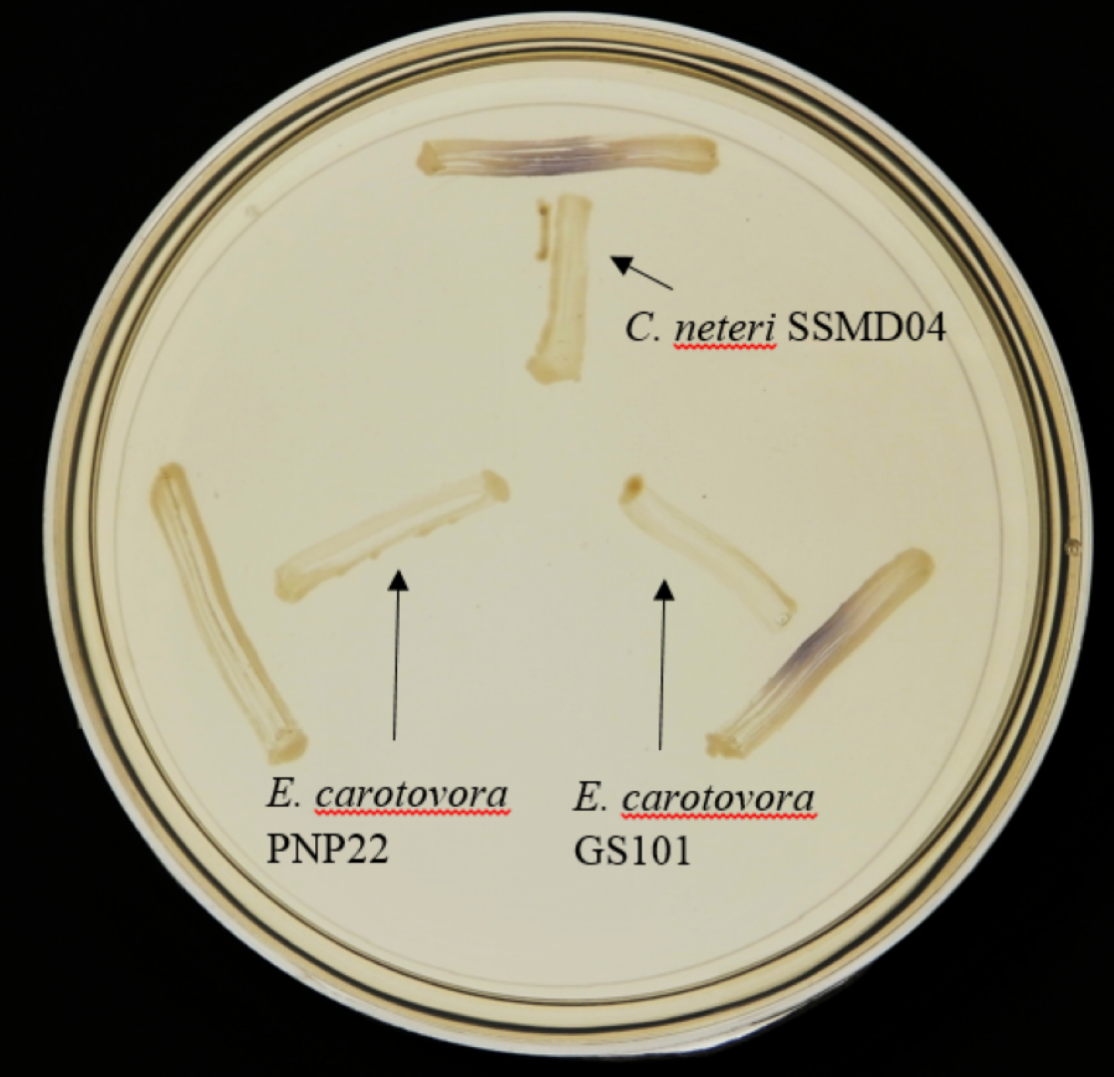
Screening for AHL-type QS activity of *C. neteri* SSMD04 using the biosensor *C. violaceum* CV026. The positions of *C. neteri* SSMD04, *E. carotovora* PNP22, *E. carotovora* GS101 are indicated by arrows, whereas *C. violaceum* CV026 was streaked perpendicularly against the tested strain at the periphery of the plate.

Since AHL-type QS has been known to regulate virulence and food spoilage traits in some bacteria, we hypothesized that *C. neteri* exhibits QS activity as well. In order to investigate the presence of AHL-type QS activity in *C. neteri* SSMD04, it was streaked on LBA against biosensor *C. violaceum* CV026, which would show purple pigmentation due to the production of violacein in the presence of short chain AHLs. The result shown in Fig. 2 indicated the presence of exogenous AHLs in *C. neteri* SSMD04 culture. *E. carotovora* GS101, a known AHL producing *Erwinia* strain, and *E. carotovora* PNP22, an AHL-synthase mutant were chosen as positive and negative controls, respectively.

**Figure 3:**
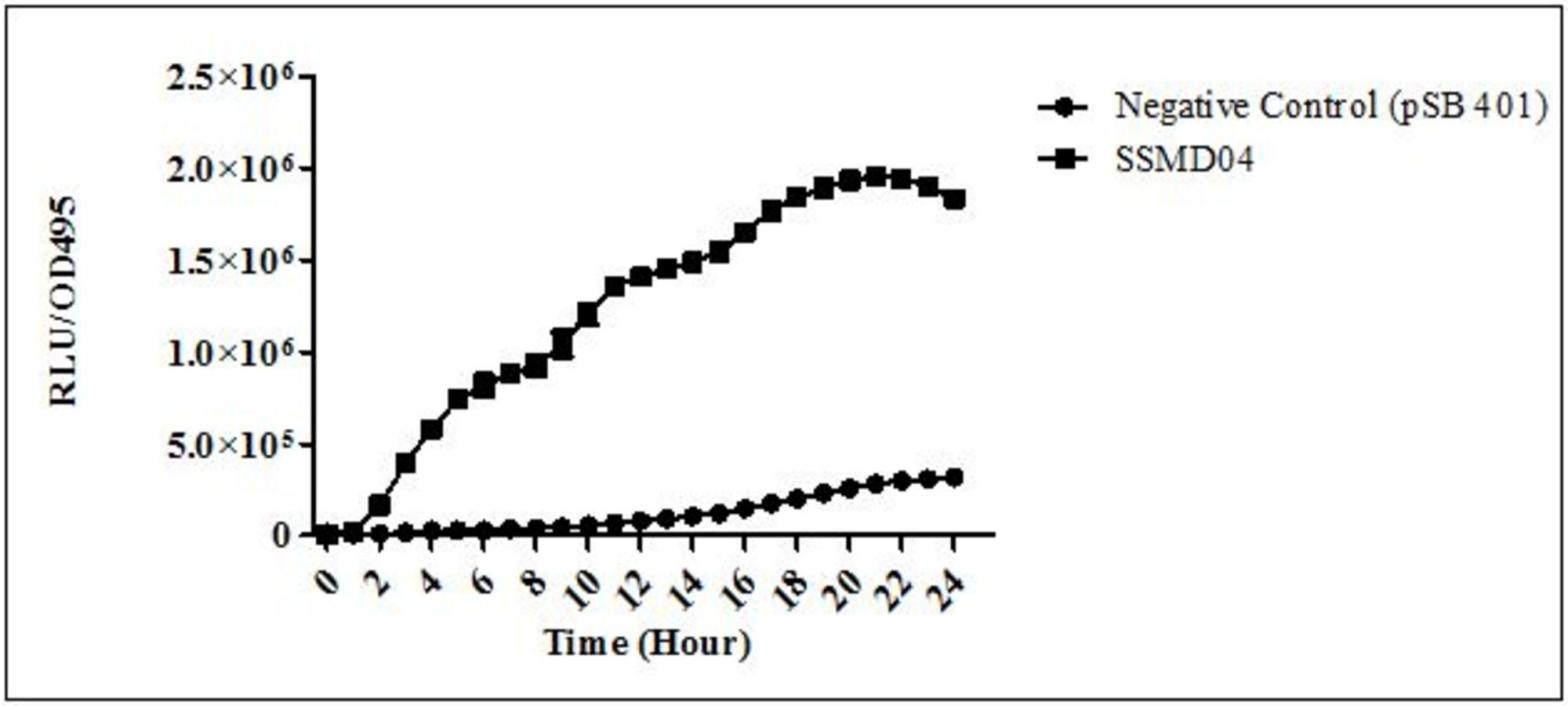
Detection of AHL by *E. coli* (pSB401). Relative light unit (RLU)/OD495 against incubation time of cultures of *E. coli* (pSB401) in the presence of extracted AHLs (square plots) and negative control (circle plots).

*C. neteri* SSMD04 also activated *lux*-based biosensor *E. coli* (pSB401) which produces bioluminescence in the presence of short chain AHLs (Fig. 3).

### AHL identification by triple quadrupole LC/MS

**Figure 4:**
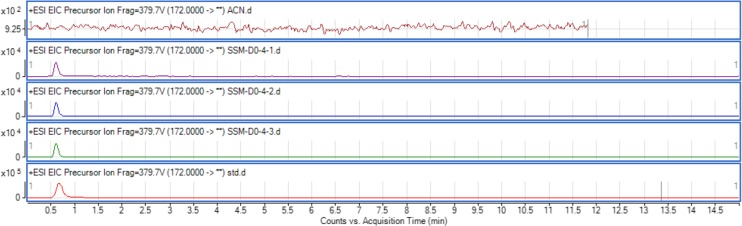
EIC of *C. neteri* SSMD04 extract. The data represented three replicates of the extract against synthetic C4-HSL, labelled as “std.” ACN was used as blank.

**Figure 5:**
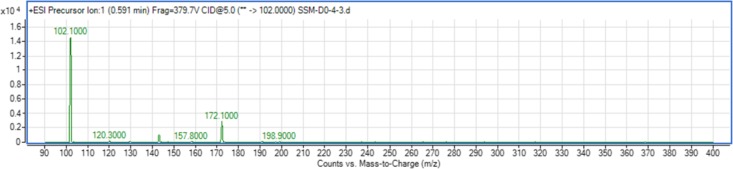
Mass spectrometry analysis of *C. neteri* SSMD04 spent supernatant extract. Product ions of the peak seen which shows that the extract of *C. neteri* SSMD04 contains C4-HSL.

The use of both biosensors (*C. violaceum* CV026 and *E. coli* (pSB401)) does not give information on the specific type of AHL produced since each detects a variety of AHLs. Therefore, triple quadrupole LC/MS was employed in the identification of the AHL produced by *C. neteri* SSMD04. The extracted-ion chromatogram (EIC) generated from the triple quadrupole LC/MS system showed a peak with the same retention time as that of the synthetic *N*-butyryl-homoserine lactone (C4-HSL) standard, which was constantly present in all three replicates (Fig. 4). Analysis of the mass spectrum (MS) data revealed the presence of a peak with mass-to-charge ratio (*m*/*z*) of 172 (Fig. 5), which is consistent with the previously reported value (Ortori et al., 2011). This implication was strengthened by the presence of a product ion peak (*m*/*z* = 102), confirming the presence of a homoserine lactone ring, the invariant structure of AHLs. This result is in agreement with the results of the biosensor strains, *C. violaceum* CV026 and *E. coli* (pSB401), as both respond to C4-HSL.

### Lipase activity

**Figure 6:**
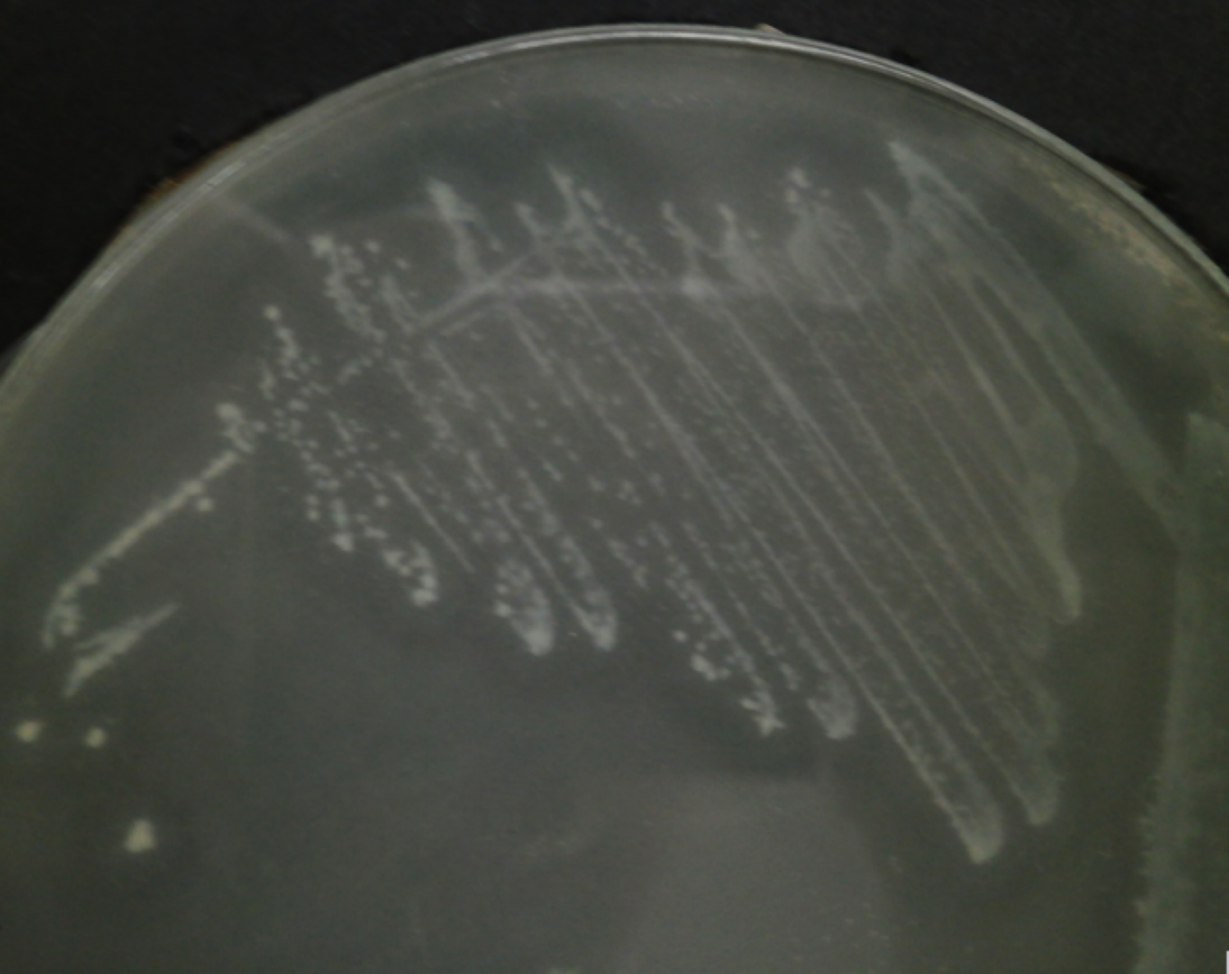
*C. neteri* SSMD04 grown on medium containing 0.5% corn oil. The halo zone around the colonies indicate lipase activity.

*C. neteri* SSMD04 was streaked on medium containing 0.5% (v/v) corn oil and incubated overnight to test for its lipase activity. The halo zone was visible around the colonies after 24 h of incubation, but it was more visible after 48 h of incubation (Fig. 6).

### *luxI/R* homologues search and analysis

From the data generated by NCBI prokaryotic genome annotation pipeline, a 636 bp *luxI* homologue, hereafter named *cneI*, was found in the genome. This gene shares 70% base pair similarity with *N*-acyl homoserine lactone synthase *croI* of *Citrobacter rodentium* ICC168. Analysis of amino acid sequence of *cneI* using InterPro (Mitchell et al., 2015) identified the presence of an acyl-CoA-*N*-acyltransferase domain, the structural domain of *N*-acyl homoserine lactone synthetases (Gould, Schweizer & Churchill, 2004; Watson et al., 2002).

Eight bp away from *cneI* is a sequence encoding a hypothetical protein, potentially the cognate receptor, a *luxR* homologue (*cneR*). The coding region was found to be 705 bp long and share 70% similarity with *croR* of *C. rodentium*. Analysis of this protein reveals two signature domains, the autoinducer-binding domain and the C-terminal effector.

**Figure 7:**
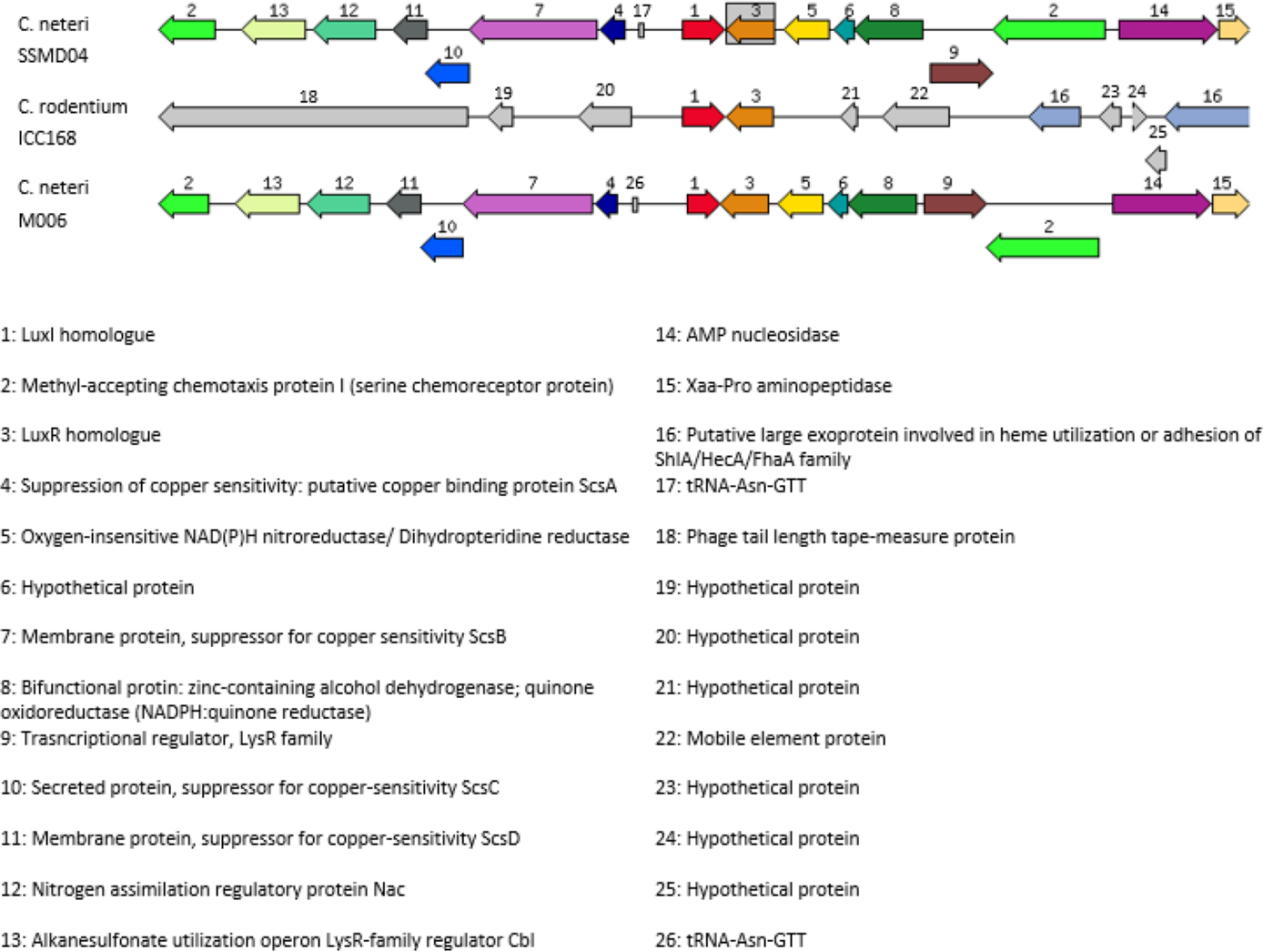
Schematic representation of *cneI/R* locus of *C. neteri* SSMD04 in comparison with *C. neteri* M006 and *C. rodentium* ICC168 (GenBank ID: CP0009458.1 and FN543502.1, respectively). Species and strain are shown at the left. Each gene is numbered and labelled accordingly. Homologous genes are represented in the same colour. The arrows show the orientation of the gene. Overlapping genes are arranged below the line.

In order to investigate the relatedness of *cneI/R* and *croI/R*, the organization of *cneI/R* and their flanking region was examined and comparison to another *C. neteri* genome, M006, as well as *C. rodentium* ICC168 was made (Fig. 7). As can be seen, gene organization of *cneI/R* and their flanking region is highly similar in both *C. neteri* strains, but displays no similarity with *croI/R* and their flanking region. Interestingly, the genome of *C. davisae* DSM 4568 harbours no *luxI/R* homologous pair, suggesting that AHL-type quorum sensing is not present in all members of the genus *Cedecea*.

**Figure 8:**
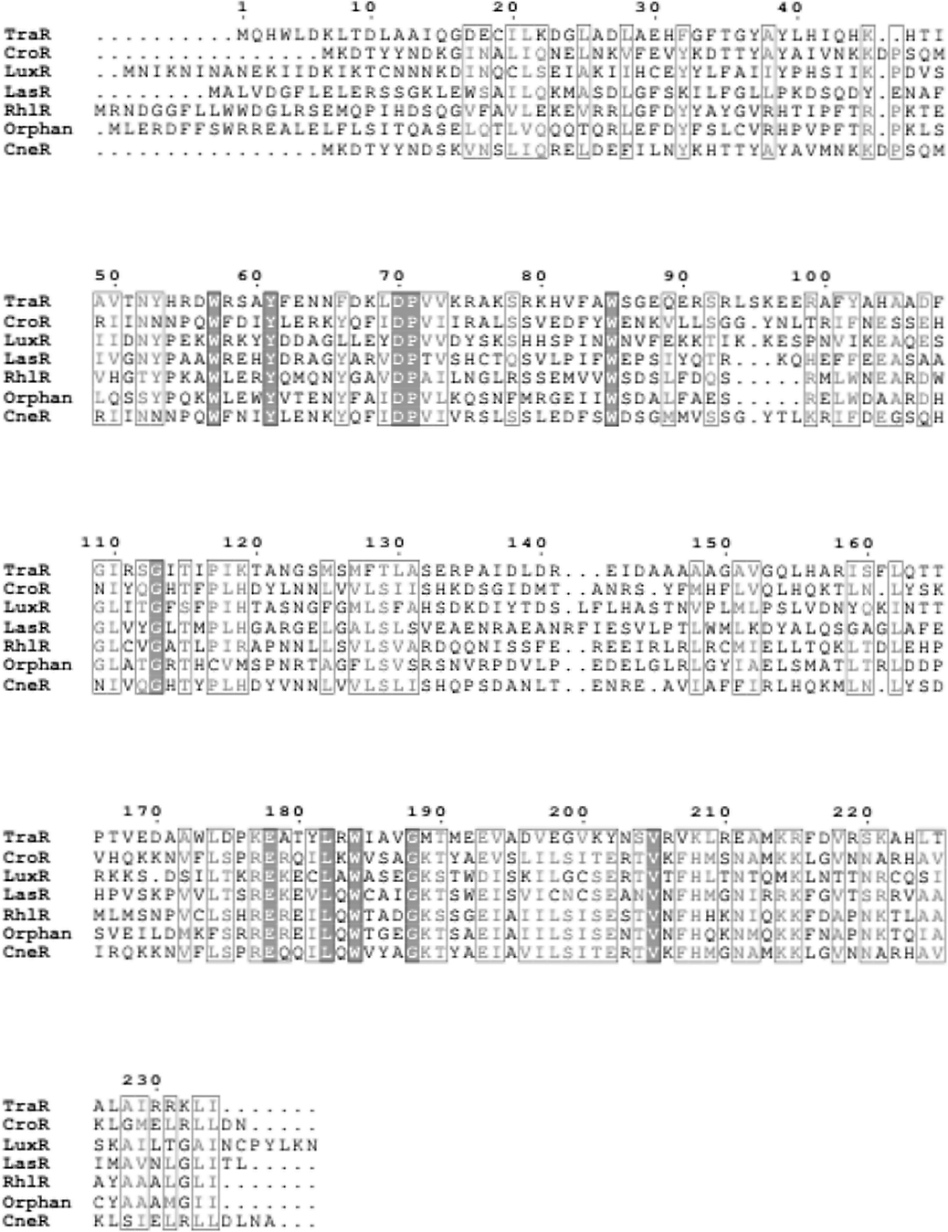
Multiple sequence alignment of CneR, *C. neteri* SSMD04 orphan LuxR (Orphan) with five other canonical QS LuxR-type proteins. TraR, CroR, LuxR, LasR, and RhlR (GenBank ID: 282950058, 59482356, 299361, 541657, 1117981, 740696391, 689266442, respectively) are LuxR-type proteins involved in quorum sensing from *A. tumefaciens*, *C. rodentium*, *Vibrio fischeri*, *Pseudomonas aeruginosa* (LasR and RhlR), respectively. Identical residues are denoted by a vertical filled bar, and conserved residues are denoted by unfilled box. TraR residue numbering is shown above the alignment as reference.

Apart from that, a sequence potentially encoding an orphan *luxR* type receptor (723 bp) was also found within the genome. This luxR homologue shares 69% sequence homology to *luxR* homologue of *Enterobacter asburiae* L1. Multiple sequence alignment of this orphan LuxR, CneR, and other canonical LuxR-type proteins (Fig. 8) reveals the presence of conserved sites, residues 57, 61, 70, 71, 85, 113, 178, 182, 188 (TraR sequence numbering used as a reference), which are present in at least 95% of LuxR-type proteins (Whitehead et al., 2001; Zhang et al., 2002).

**Figure 9:**
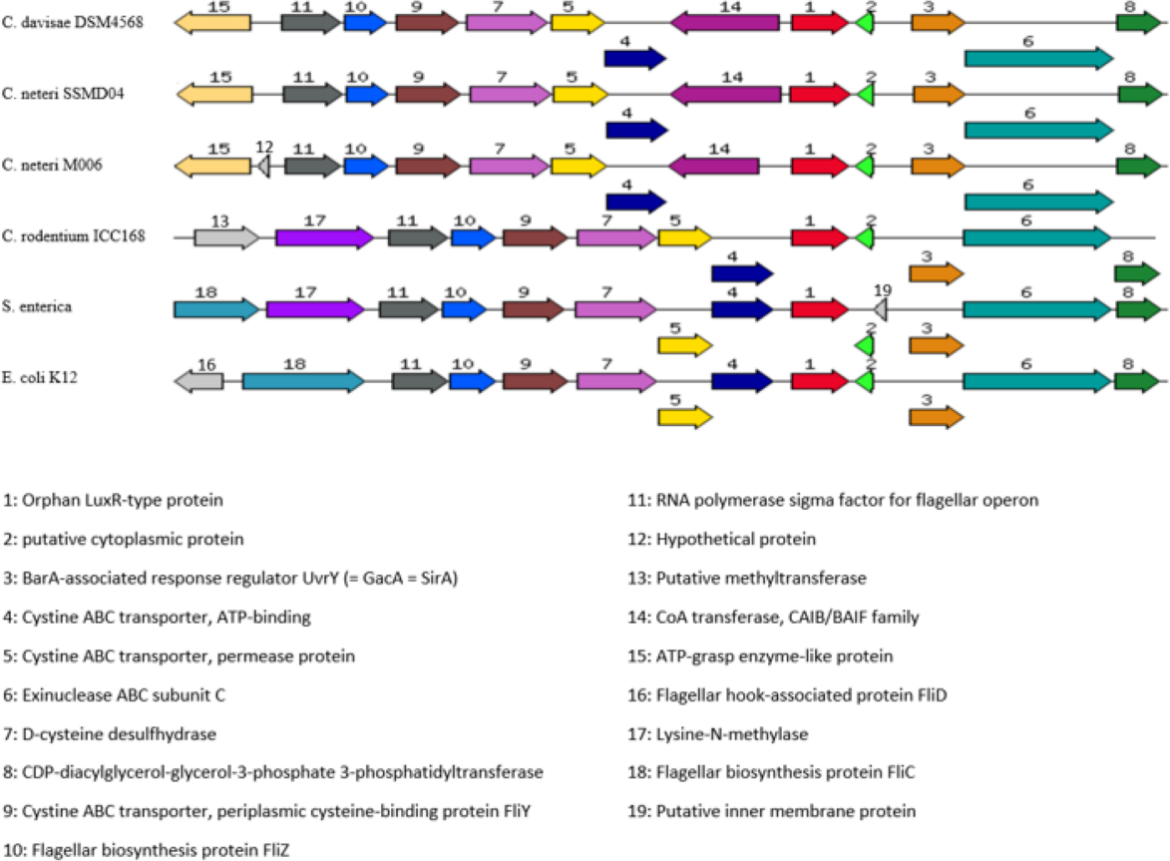
Organization of *C. neteri* SSMD04 orphan *luxR* and its flanking genes in comparison with other selected species *C. davisae* DSM4568, *C. neteri* M006, *C. rodentium* ICC168, *Salmonella enterica* subsp. *enterica* serovar Ch. Organization of *C. neteri* SSMD04 orphan *luxR* and its flanking genes in comparison with other selected species *C. davisae* DSM4568, *C. neteri* M006, *C. rodentium* ICC168, *Salmonella enterica* subsp. *enterica* serovar Choleraesuis str. SC-B67 and *E. coli* K12 (GenBank ID: 513473511, 690276415, 282947233, 62178570, 556503834, respectively).

Orphan LuxR is known to be present in *E. coli* and *Salmonella* which allows response to exogenous AHLs (Ahmer, 2004). The function of the orphan LuxR in *C. neteri* SSMD04 is not known. However, genome comparison of *C. neteri* SSMD04 and M006, *C. davisae* DSM4568, *C. rodentium* ICC168, *S. enterica* subsp. *enterica* serovar Choleraesuis str. SC-B67, and *E. coli* K12 shows a considerable degree of conservation (Fig. 9). This is especially true for the 3 genomes of the genus *Cedecea*, despite the absence of canonical *luxI/R* pair in *C. davisae* DSM4568 genome.

## Discussion

*Cedecea* spp. are not well studied. Despite the evidence of their ability to infect human, their medical significance can be overlooked due the poor understanding of their physiology and etiology. Besides that, they are potentially challenging pathogens due to their resistance towards a wide range of antimicrobial agents, such as cephalothin, extended spectrum cephalosporins, colistin, and aminoglycosides (Mawardi et al., 2010; Abate, Qureshi & Mazumder, 2011; Dalamaga et al., 2008). To date, isolation of *C. neteri* from a non-clinical source has not been reported. Little is known about the mechanism of pathogenesis in this bacterium, and it has never been reported to be present in food. Nevertheless, the isolation of *C. neteri* SSMD04 from a food source expands the current knowledge on diversity of the genus *Cedecea*.

Although employed by a wide range of Gram-negative bacteria in gene regulation that allows the alteration of behaviour on a population level (Waters & Bassler, 2005), AHL-type QS activities have not been reported in *C. neteri*. Some bacteria utilizes QS to regulate virulence and thus gaining advantage of expressing virulence factors only when the population density is large enough to triumph the hosts’ immune system (Passador et al., 1993; Brint & Ohman, 1995; McClean et al., 1997; Thomson et al., 2000; Weeks et al., 2010). Regulation of virulence factors by QS has been extensively studied in *Pseudomonas aeruginosa*. The transcriptional regulator LasR in *P. aeruginosa*, a homologue of LuxR regulates expression of virulence genes such as *lasB*, *lasA*, *aprA*, and *toxA* in the presence of *N*-(-3-oxododecanoyl)-L-homoserine lactone (OC12-HSL), synthesized by LasI, a homologue of LuxI (Gambello & Iglewski, 1991; Gambello, Kaye & Iglewski, 1993; Pearson et al., 1994; Toder, Gambello & Iglewski, 1991). QS regulated virulence can also be seen in other bacteria such as *Burkholderia cepacia*, *E. carotovora*, and *Agrobacterium tumefaciens* (De Kievit & Iglewski, 2000). Given the understanding that *C. neteri* can act as human pathogen, it can be hypothesized that AHL-type QS activity in *C. neteri* is involved in the regulation of virulence factors. However, further studies on clinical as well as non-clinical strains would help in the solution of this hypothesis.

The whole genome sequence provides very valuable information in studying the genetic basis of QS in *C. neteri* SSMD04. The finding of *cneI/R* in this genome, lying adjacent to each other, demonstrated a common feature of *luxI/R* homologues (Brint & Ohman, 1995; Williamson et al., 2005). Analysis of the amino acid sequence of the *cneI/R* pair with InterPro agreed with their identity. The *cneI/R* pair was found to be most similar to *croI/R* in *C. rodentium*. *C. rodentium* has been found to produce C4-HSL as the major AHL and C6-HSL as the minor (Coulthurst et al., 2007). *C. neteri* SSMD04 also produces C4-HSL, but it does not produce detectable levels of C6. Nevertheless, chromosomal region comparison between two *C. neteri* genomes and *C. rodentium* shows no similarity in gene organization at the *luxI/R* homologues region. Interestingly, gene organization of the orphan *luxR* of *C. neteri* is highly similar to that of *C. rodentium* as well as *E. coli* and *Salmonella*. This probably allows *C. neteri* to interfere with AHLs produced by other bacterial species and thus improving population fitness.

The presence of lipase-positive *C. neteri* in marinated oily fish strongly suggests its role as a potential food spoilage agent, not only because of its ability to survive an extreme environment of high salinity and acidity, but also the fact that AHLs have long been associated with food spoilage via regulation of the proteolytic and lipolytic pathways (Skandamis & Nychas, 2012; Bruhn et al., 2004). Nevertheless, the roles of *C. neteri* in pathogenesis and food spoilage still require more information to be elucidated.

## Conclusion

This study has confirmed the production of C4-HSL by *C. neteri* SSMD04 isolated from *Shime saba* sashimi. This is the first report of QS activity in *C. neteri*. However, the function of QS in *C. neteri* SSMD04 is still unknown. We hope that further studies coupled with the available genome information of *C. neteri* SSMD04 can help to elucidate the regulatory circuit of *C. neteri* SSMD04 by QS.

## Supplemental Information

10.7717/peerj.1216/supp-1 Supplemental Information 1 Genome sequences

## Additional Information and Declarations

### DNA Deposition

The following information was supplied regarding the deposition of DNA sequences: We have submitted the whole genome sequence of *C. neteri* SSMD04 to NCBI: NCBI Reference Sequence: NZ_CP009451.1 GenBank: CP009451.1 GI: 689262542.
